# Blue Whiting (*Micromesistius poutassou*) Protein Hydrolysates Increase GLP-1 Secretion and Proglucagon Production in STC-1 Cells Whilst Maintaining Caco-2/HT29-MTX Co-Culture Integrity

**DOI:** 10.3390/md20020112

**Published:** 2022-01-31

**Authors:** Shauna Heffernan, Leo Nunn, Pádraigín A. Harnedy-Rothwell, Snehal Gite, Jason Whooley, Linda Giblin, Richard J. FitzGerald, Nora M. O’Brien

**Affiliations:** 1 School of Food and Nutritional Sciences, University College Cork, T12 YN60 Cork, Ireland; shauna.heffernan@umail.ucc.ie; 2 Department of Biological Sciences, Munster Technological University, T12 P928 Cork, Ireland; leo.nunn@teagasc.ie; 3 Department of Biological Sciences, University of Limerick, V94 T9PX Limerick, Ireland; padraigin.harnedy@ul.ie (P.A.H.-R.); dick.fitzgerald@ul.ie (R.J.F.); 4 Bio-Marine Ingredients Ireland Ltd., Lough Egish Food Park, A75 WR82 Castleblaney, Ireland; snehal@biomarine.ie (S.G.); jason@biomarine.ie (J.W.); 5 Teagasc Food Research Centre, Moorepark, Fermoy, P61 C996 Cork, Ireland; linda.giblin@teagasc.ie

**Keywords:** blue whiting, protein hydrolysates, satiety hormones, GLP-1, STC-1, gut health

## Abstract

Inducing the feeling of fullness via the regulation of satiety hormones presents an effective method for reducing excess energy intake and, in turn, preventing the development of obesity. In this study, the ability of blue whiting soluble protein hydrolysates (BWSPHs) and simulated gastrointestinal digested (SGID) BWSPHs, to modulate the secretion and/or production of satiety hormones, such as glucagon-like peptide-1 (GLP-1), cholecystokinin (CCK) and peptide YY (PYY), was assessed in murine enteroendocrine STC-1 cells. All BWSPHs (BW-SPH-A to BW-SPH-F) (1.0% *w*/*v* dw) increased active GLP-1 secretion and proglucagon production in STC-1 cells compared to the basal control (Krebs–Ringer buffer) (*p* < 0.05). The signaling pathway activated for GLP-1 secretion was also assessed. A significant increase in intracellular calcium levels was observed after incubation with all BWSPHs (*p* < 0.05) compared with the control, although none of the BWSPHs altered intracellular cyclic adenosine monophosphate (cAMP) concentrations. The secretagogue effect of the leading hydrolysate was diminished after SGID. Neither pre- nor post-SGID hydrolysates affected epithelial barrier integrity or stimulated interleukin (IL)-6 secretion in differentiated Caco-2/HT-29MTX co-cultured cells. These results suggest a role for BWSPH-derived peptides in satiety activity; however, these peptides may need to be protected by some means to avoid loss of activity during gastrointestinal transit.

## 1. Introduction

Excess energy intake is the main contributor to the increasing prevalence of obesity worldwide with overweight and obesity now taking fifth place in the leading causes of global death [1,2]. Researchers are currently investigating various potential obesity prevention strategies in an attempt to manage the global obesity pandemic and to alleviate the pressure that obesity and obesity-related diseases place on our healthcare systems. Inducing the feeling of fullness through the regulation of hormonal signaling presents an effective method for reducing food intake and, in turn, preventing the onset of obesity [3]. Satiety hormones which can be categorized as either long-term or short-term regulators, inform the brain about fluctuations of body mass and communicate the energy available in the gastrointestinal tract, respectively [4]. Numerous weight loss drug-therapies, including satiety hormone analogues, are available commercially [5], however, the identification of non-pharmacological satiating components from food sources may present cost-effective, safe alternatives to synthetic drugs for weight management.

The fish species blue whiting (*Micromesistius poutassou*) has been landed in high volumes in the Northeast Atlantic recently due to the Common Fisheries Policy reform (EU 1380/2013, Article 15) which introduced a landing obligation for all commercial fisheries. As there is a limited number of species that dominate seafood consumption, the majority of blue whiting is processed into fishmeal and oil. The opportunity now exists to up-value this underutilised source of high-quality protein through the identification of health-enhancing protein fractions with potential applications as high-value functional food ingredients. Several marine-derived peptide mixtures and hydrolysates are commercially available worldwide as anti-obesity food supplements, many of which contain a large variety of non-identified or partially identified peptides [6]. Blue whiting muscle protein hydrolysates have previously been shown to modulate the secretion of satiety hormones in vitro and in vivo, associated with a subsequent reduction in food intake and body weight gain [7]; however, utilisation of the whole fish as the starting material for bioactive peptide production would reduce the yield of fish by-products, presenting environmental benefits. There are considerable advantages to identifying bioactive proteins from blue whiting fish for application as functional food ingredients, such as (i) the discovery of a potential natural therapeutic approach for disease prevention, in place of or in combination with reduced doses of conventional synthetic drugs, (ii) managing environmental sustainability through exploiting abundant, underutilised blue whiting fish stocks, and (iii) promoting commercial sustainability through converting low-value blue whiting to high-value proteinaceous ingredients. However, the development of functional foods is faced with many challenges and is dependent on (i) cost-effective generation of bioactive compounds, (ii) identification of the level of bioactive compound required to reach target cells to exert their bioactive effect, taking potential degradation during digestion and absorption into account and (iii) safety of the final product [6].

The gastrointestinal tract is the site of nutrient digestion and absorption and plays a key role in mediating the physiological effects induced by ingested nutrients as it represents the largest endocrine organ in the body [8]. It is generally accepted that enteroendocrine cells, which are found scattered along the epithelial layer of the gastrointestinal tract, are characterised by the hormones they secrete, i.e., I cells secreted cholecystokinin (CCK) and L cells secreted glucagon-like peptide-1 (GLP-1) and peptide YY (PYY); however, a study by Haber et al. (2017) observed an unexpected degree of heterogeneity in hormone expression from specific enteroendocrine cells [9]. With that being said, gut hormone expression does vary along the gastrointestinal tract with CCK predominantly expressed in the duodenum and jejunum, GLP-1 in the jejunum, ileum, and colon, and PYY in the distal ileum and colon [10]. These hormones are secreted basolaterally in the gut barrier upon exposure of enteroendocrine cells to digested nutrients to modulate physiological responses, including gastric emptying, gut mobility, and central nervous system signaling. Although a heterogenous cell line, STC-1 cells which are derived from murine enteroendocrine tumours, are capable of secreting satiety hormones including CCK, PYY and GLP-1, thus are a popular cellular model for the assessment of nutrient-stimulating hormone release [11]. 

The GLP-1 hormone, which is generated as a result of post translational modifications during proglucagon processing, has received significant attention due to its classification as both an anorexigenic hormone and an incretin hormone [12]. Fish protein hydrolysates, which modulated plasma GLP-1 levels have demonstrated anti-hyperglycemic and satiating effects in clinical trials [13] and have been shown to induce GLP-1 secretion in vitro through modulating intracellular levels of cyclic adenosine monophosphate (cAMP) and/ or Ca^2+^ [14]. Nutrient-induced GLP-1 secretion may occur in response to increased intracellular levels of cAMP and/ or Ca^2+^ through activation of G-protein coupled receptors or nutrient transporters via enteroendocrine cell membrane depolarisation [15]. Although GLP-1 is produced in its active forms (GLP-1_(7–36)_ amide and GLP-1_(7–37)_), the lifespan of active GLP-1 is limited with a circulation half-life of less than 2 min due to cleavage by the endogenous proteolytic enzyme dipeptidyl peptidase IV (DPP-IV) to its inactive forms (GLP-1_(9–36)_ amide or GLP-1_(9–37)_) [16]. Therefore, not only can food components promote GLP-1 hormone signaling through increasing the production/ release of GLP-1, or activating GLP-1 receptors, but also by increasing hormone circulation time via DPP-IV inhibition. 

Protein and hydrolysed protein fractions are reported to induce greater satiating effects than carbohydrates and fats [17]. Satiating proteins have been identified in numerous food sources including plants, pulses, eggs, dairy, seafood and meat [18]; however, their digestion, bioavailability and metabolism have not been studied sufficiently [6]. In addition to proteases in the gut lumen, proteins/ hydrolysates/ peptides can be further degraded by brush border membrane proteases or blood plasma proteases, resulting in physicochemical changes which can alter their bioactive potential. Moreover, it is important that bioactive protein digestion products which target gut cells do not cause inflammation or damage to the gut barrier.

The research presented herein is a continuation of a study by Harnedy-Rothwell et al. (2021) in which blue whiting (*Micromesistius poutasso*) soluble protein hydrolysates (BWSPHs) were prepared using food-grade microbial proteases, and assessed in terms of their amino acid profile and physicochemical properties. In addition, all BWSPHs tested mediated DPP-IV inhibitory (IC_50_: 2.12–2.90 mg protein/mL) and insulin secretory activity (2.5 mg/mL; 4.7 to 6.4-fold increase compared to the basal control (5.6 mM glucose alone)) [19]; therefore, the present study was designed to assess the ability of BWSPHs to modulate the secretion and/or production of the satiety hormones GLP-1, CCK and PYY in STC-1 cells. Additionally, the impact, if any, that simulated gastrointestinal digestion (SGID) has on this activity was studied, as well as the mechanism by which the leading hydrolysate mediates hormonal signaling. Finally, the effect of SGID BWSPHs on intestinal barrier integrity and inflammation, using a 21-day-old differentiated Caco-2/HT-29MTX monolayer, as a model of a healthy gut barrier was investigated.

## 2. Results

### 2.1. BWSPHs Have No Effect on Cell Viability

The cytotoxicity of six BWSPHs (BW-SPH-A to BW-SPH-F) and six SGID BWSPHs (BW-SPH-A-GI to BW-SPH-F-GI) (0–1.0% *w*/*v* dw) in STC-1 cells (Supplementary Table S1) and a Caco-2/HT-29MTX co-cultured cells (Supplementary Table S2) after 24 h incubation, was studied. There was no significant difference in the viability of cells exposed to BWSPHs (0–1.0% *w*/*v* dw) compared with the control (DMEM) indicating the non-toxic effect of BWSPHs and their corresponding simulated gastrointestinal digests in STC-1 cells and the co-cultured cells (Supplementary Tables S1 and S2). Based on these results, a protein hydrolysate concentration of 1.0% *w*/*v* dw was chosen for all cellular assays. 

### 2.2. BWSPHs Do Not Induce Inflammation or Disrupt Co-Culture Integrity

**Figure 1:**
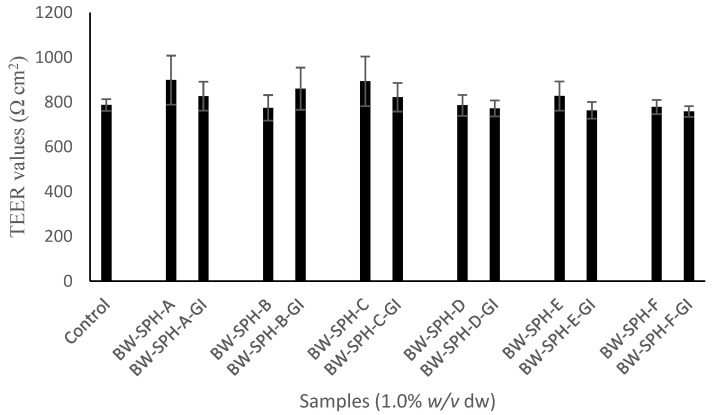
Transepithelial electrical resistance (TEER) values of 21-day-old differentiated Caco-2/HT-29MTX monolayers treated with blue whiting (*Micromesistius poutassou*) soluble protein hydrolysates before (BW-SPH-A to BW-SPH-F) and after simulated gastrointestinal digestion (BW-SPH-A-GI to BW-SPH-F-GI) for 4 h. The control is Hank’s Balanced Salt Solution (HBSS) only. Data are expressed as mean ± SEM of three independent experiments. Significance was measured using ANOVA followed by Dunnett’s test.

After 21 days, cell monolayers were considered to have reached confluence and were fully polarised with TEER values exceeding 700 Ω·cm^2^. As shown in Figure 1, BWSPHs and SGID BWSPHs did not alter cell monolayer integrity, with TEER values ranging from 774.3 ± 57.2–898.3 ± 109.3 Ω·cm^2^ and 758.0 ± 23.7–860.0 ± 94.3 Ω·cm^2^, respectively, compared with the HBSS buffer control (787.0 ± 26.2 Ω·cm^2^) after 4 h exposure. An inflammatory effect of BWSPHs and their corresponding gastrointestinal digests on human intestinal epithelial cells was investigated by measuring the reputable pro-inflammatory cytokine, interleukin (IL)-6 in the apical compartment of Caco-2/HT-29MTX co-cultured cells which were exposed to the test samples for a 4 h period. IL-6 concentrations in the co-culture apical compartments containing BWSPHs, SGID BWSPHs or HBSS buffer were below the minimum limit of detection (4 pg/mL), indicating that these protein hydrolysates could not induce an IL-6 response upon contact with gut epithelial cells.

### 2.3. BWSPHs Increase Proglucagon Production and GLP-1 Secretion from STC-1 Cells

**Figure 2:**
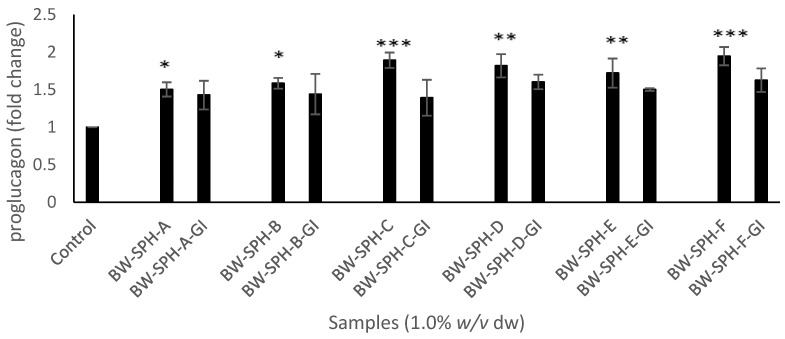
Levels of proglucagon mRNA transcripts in STC-1 cell lysates after exposure to blue whiting protein hydrolysates before (BW-SPH-A to BW-SPH-F) and after simulated gastrointestinal digestion (BW-SPH-A-GI to BW-SPH-F-GI) for 4 h in Krebs–Ringer buffer. *36B4* (RPLP0) was used as a reference gene. Data are expressed as mean ± SEM of three independent experiments. Significance was measured using ANOVA followed by Dunnett’s test. * denotes statistical significance between individual samples and the control (Krebs–Ringer buffer) (*p* < 0.05), ** *p* < 0.01, *** *p* < 0.001.

To determine the potential of BWSPHs to modulate satiety, their ability to produce satiety hormones at a transcriptional level and increase active GLP-1 secretion in murine enteroendocrine STC-1 cells was assessed. GLP-1 precursor proglucagon mRNA transcript levels were upregulated upon exposure of STC-1 to BWSPHs for 4 h compared with the negative control (Krebs–Ringer buffer) (*p* < 0.05) (Figure 2). However, post-SGID BWSPHs (BW-SPH-A-GI to BW-SPH-F-GI) did not alter proglucagon production significantly (*p* > 0.05) compared to the basal control. In addition, no significant differences were observed between proglucagon levels produced by BWSPHs before SGID and after SGID (Figure 2). 

**Figure 3:**
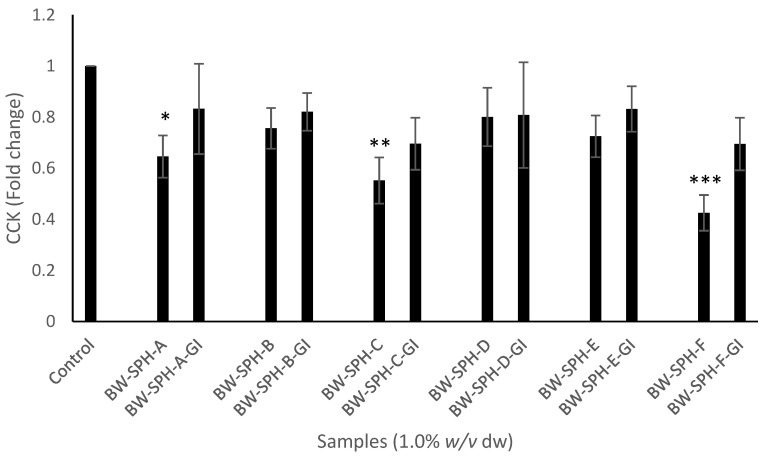
Levels of cholecystokinin (CCK) mRNA transcripts in STC-1 cell lysates after exposure to blue whiting protein hydrolysates before (BW-SPH-A to BW-SPH-F) and after simulated gastrointestinal digestion (BW-SPH-A-GI to BW-SPH-F-GI) for 4 h in Krebs–Ringer buffer. *36B4* (RPLP0) was used as a reference gene. Data are expressed as mean ± SEM of three independent experiments. Significance was measured using ANOVA followed by Dunnett’s test. * denotes statistical significance between individual samples and the control (Krebs–Ringer buffer) (*p* < 0.05), ** *p* < 0.01, *** *p* < 0.001.

**Figure 4:**
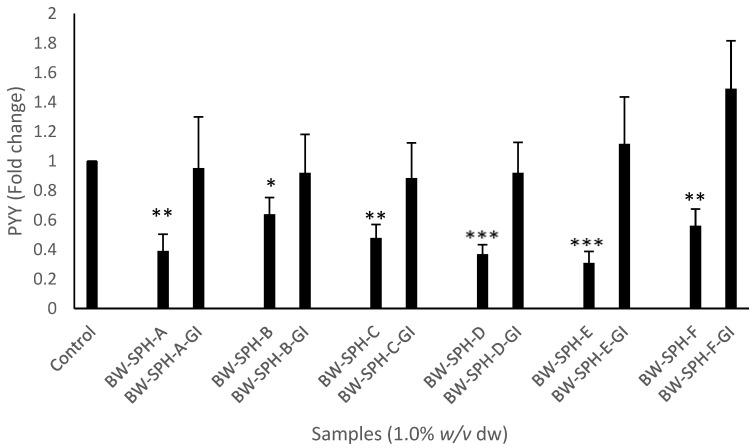
Levels of peptide YY (PYY) mRNA transcripts in STC-1 cell lysates after exposure to blue whiting protein hydrolysates before (BW-SPH-A to BW-SPH-F) and after simulated gastrointestinal digestion (BW-SPH-A-GI to BW-SPH-F-GI) for 4 h in Krebs–Ringer buffer. *36B4* (RPLP0) was used as a reference gene. Data are expressed as mean ± SEM of three independent experiments. Significance was measured using ANOVA followed by Dunnett’s test. * denotes statistical significance between individual samples and the control (Krebs–Ringer buffer) (*p* < 0.05), ** *p* < 0.01, *** *p* < 0.001.

Hydrolysates BW-SPH-A, BW-SPH-C and BW-SPH-F reduced CCK mRNA transcript levels compared to the basal control (*p* < 0.05), whereas BW-SPH-B, BW-SPH-D and BW-SPH-E induced no effect on CCK production after the 4 h incubation period in STC-1 cells (*p* > 0.05) (Figure 3). In addition, all BWSPHs reduced PYY mRNA transcript levels compared with the Krebs–Ringer buffer control (*p* < 0.05) (Figure 4). The CCK and PYY mRNA transcript levels observed after STC-1 cell exposure to SGID BWSPHs were not significantly different to the control (*p* > 0.05) (Figure 3 and Figure 4). In addition, no significant differences were observed between CCK and PYY levels produced by BWSPHs before SGID and after SGID (*p* > 0.05) (Figure 3 and Figure 4).

**Figure 5:**
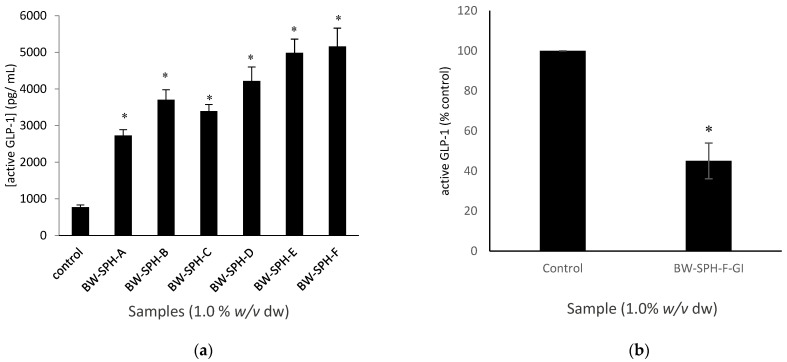
(**a**) Secretion of active glucagon-like peptide-1 (GLP-1) from STC-1 cells (0.625 × 10^6^ cells/well) exposed to blue whiting (*Micromesistius poutassou*) soluble protein hydrolysates (BWSPHs) at 1.0% (*w*/*v* dw) or Krebs–Ringer buffer (control) for 4 hrs. * denotes statistical significance between individual samples and the control, measured using ANOVA followed by Dunnett’s test (*p* < 0.05). (**b**) Secretion of active GLP-1 from STC-1 cells (0.625 × 10^6^ cells/well) exposed to BWSPH BW-SPH-F post simulated gastrointestinal digestion (SGID) (BW-SPH-F-GI) at 1.0% (*w*/*v* dw) or Krebs–Ringer buffer (control) for 4 h, expressed as a percentage of the control. * indicates a significant difference between the control and BW-SPH-F-GI values measured by Student’s *t*-test (*p* < 0.05). Data are expressed as mean ± SEM of three independent experiments.

Following a 4 h incubation period, all BWSPHs tested increased the secretion of active GLP-1 in STC-1 cells compared to the basal control (Krebs–Ringer buffer) (*p* < 0.05) (Figure 5a). The concentration of active GLP-1 secreted from STC-1 cells exposed to BWSPHs ranged from 2727.6 ± 160.5 pg/mL (BW-SPH-A) to 5163.8 ± 495.6 pg/mL (BW-SPH-F), with Krebs–Ringer buffer only stimulating the secretion of 769.7 ± 63.5 pg/mL active GLP-1 from STC-1 cells (Figure 5a). As active GLP-1 concentrations were highest in cells treated with undigested hydrolysate BW-SPH-F, SGID hydrolysate BW-SPH-F-GI was chosen to assess if in vitro digestion influenced BWSPH’s active GLP-1 stimulating ability. The GLP-1 stimulating ability of BW-SPH-F was lost following SGID as active GLP-1 concentrations in STC-1 cells exposed to BW-SPH-F-GI were significantly lower than the Krebs–Ringer buffer control (*p* < 0.05) (Figure 5b). 

### 2.4. Effects of BWSPHs on GLP-1 Signaling Mechanisms

**Figure 6:**
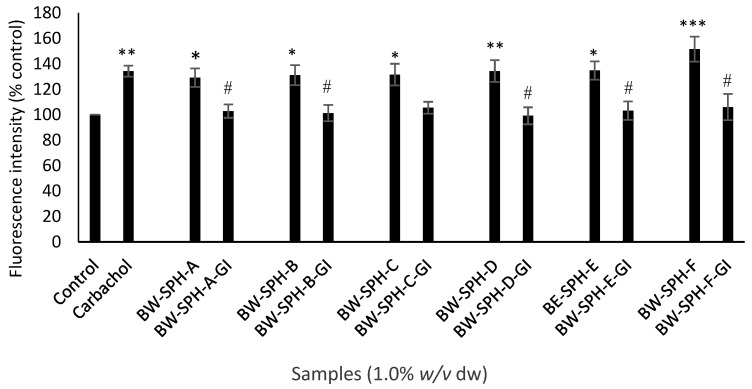
Intracellular Ca^2+^ levels in STC-1 cells (0.25 × 10^6^ cells/ mL, 200 µL/ well) after 4 h exposure to blue whiting soluble protein hydrolysates (BWSPHs, BW-SPH-A to BW-SPH-F) (1.0% *w*/*v* dw) and simulated gastrointestinal digested (SGID) BWSPHs (BW-SPH-A-GI to BW-SPH-F-GI) (1.0% *w*/*v* dw), and controls (positive control was carbachol (1 mM) and negative control was Krebs–Ringer buffer), followed by 1 h incubation with 5 µM Fluo-4-AM dye. Data are presented as intensity of fluorescence signals at excitation 494 nm and emission 506 nm compared with the control (Krebs–Ringer buffer). Significance was measured using ANOVA followed by Dunnett’s test. *, ** and *** denotes statistical significance between samples and the control at *p* < 0.05, *p* < 0.01 and *p* < 0.001, respectively. # indicates a significant difference at *p* < 0.05 between pre- and post-SGID values measured by Student’s *t*-test.

**Figure 7:**
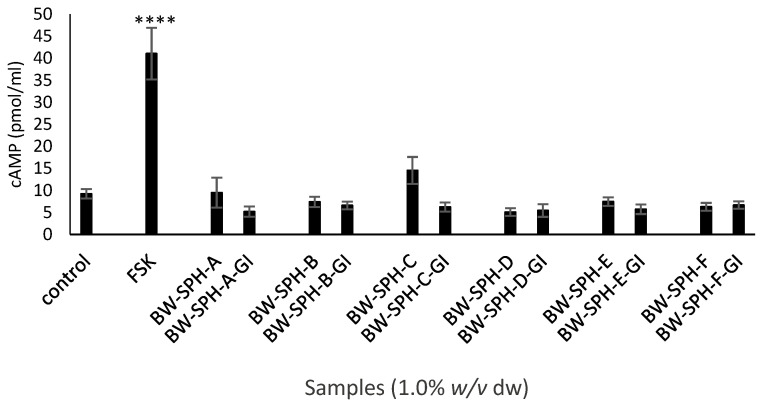
Intracellular cyclic adenosine monophosphate (cAMP) levels in STC-1 cells after 4 h exposure to blue whiting soluble protein hydrolysates (BWSPHs, BW-SPH-A to BW-SPH-F) (1.0% *w*/*v* dw) and simulated gastrointestinal digested (SGID) BWSPHs (BW-SPH-A-GI to BW-SPH-F-GI) (1.0% *w*/*v* dw), and controls (positive control was forskolin (FSK) (10 µM) and negative control was Krebs–Ringer buffer). Test samples and controls were supplemented with 1 mM 3-isobutyl-1-methylxanthine (IBMX). Data are expressed as mean ± SEM of three independent experiments. Significance was measured using ANOVA followed by Dunnett’s test. **** denotes statistical significance between samples and the negative control (*p* < 0.0001).

The effect of BWSPHs BW-SPH-A to BW-SPH-F and their simulated gastrointestinal digests (BW-SPH-A-GI to BW-SPH-F-GI) on electrogenic (Ca^2+^) and the electroneutral (cAMP) governed release of GLP-1 was assessed in STC-1 cells. Both the positive control carbachol (1 mM) and the pre-SGID BWSPHs (1.0% *w*/*v*) increased intracellular Ca^2+^ levels, compared with the basal control (Krebs–Ringer buffer) after 4 h exposure to STC-1 cells (*p* < 0.05) (Figure 6). However, post-SGID, all hydrolysates failed to elicit intracellular Ca^2+^ changes in STC-1 cells compared to the control, and fluorescence intensities were significantly lower in SGID hydrolysates compared to their undigested forms (Figure 6). Neither the pre- nor post-SGID BWSPHs induced any effect on intracellular cAMP levels compared to the control (Krebs–Ringer buffer supplemented with 1 mM IBMX) in contrast to the positive control, FSK (10 µM), which increased cAMP levels 4-fold (Figure 7). No differences in intracellular cAMP concentration were observed between the pre- and post-SGID BWSPHs after statistical analysis with Student’s t-test (Figure 7). 

## 3. Discussion

Bioactive peptides identified from low-value fish species, such as blue whiting, may represent profitable functional food ingredients due to their natural availability, low-cost extraction methods and ability to exert various health-enhancing activities. The shortcomings (time, safety, extraction efficiency) associated with traditional biomolecule extraction techniques, such as acid, alkaline, salt and solvent extraction, has led to the popular use of safer, more efficient extraction methods including enzymic hydrolysis for the effective recovery of desirable bioactive components [20]. The BWSPHs tested herein were generated from the same source material using different food-grade proteolytic enzymes and enzymatic hydrolysis conditions in a biorefinery approach at an industrial scale [19]. These BWSPHs have previously demonstrated in vitro anti-diabetic activity [19], and the results of this study show that BWSPHs may also exhibit satiating potential through synthesising the GLP-1 precursor peptide, proglucagon, and stimulating the secretion of active GLP-1 (3.5-fold for BW-SPH-A to 6.7-fold for BW-SPH-F) in STC-1 cells, indicating BWSPHs can regulate GLP-1 at both transcriptional and post-transcriptional levels. However, the GLP-1 stimulating effect of BWSPH subjected to SGID was lost indicating its instability during gut transit. The study by Harnedy-Rothwell et al. (2021) demonstrated that all pre-SGID BWSPHs inhibited the activity of DPP-IV enzyme, an enzyme responsible for inactivating more than 80% of secreted GLP-1, by 50% at concentrations ranging from 2.12–2.90 mg protein/ mL [19]. However, a significant reduction in DPP-IV inhibitory activity was observed following SGID of BW-SPH-B, -D, -E and -F (*p* < 0.05), possibly relating to the loss of bioactivity demonstrated by SGID BWSPH BW-SPH-F-GI in this study. In addition, BWSPHs subjected to in vitro gastrointestinal digestion (2.5 mg/mL) were shown to stimulate insulin secretion from clonal pancreatic BRIN-BD11 cells compared with the control (buffer/media containing 5.6 mM glucose) (*p* < 0.05), although a significant reduction in insulinotropic potency was observed following SGID of BW-SPH-F (*p* < 0.05) [19]. As GLP-1 potentiates glucose-stimulated insulin secretion, the loss of the hydrolysates secretagogue ability post-SGID may have been associated with this reduction in insulin secretion. It is well known that due to the crude nature of hydrolysate mixtures, the content of low molecular weight peptides or free amino acids may directly influence hydrolysate bioactivities. Hydrolysate composition is highly influenced by the protein source, the method of hydrolysis, hydrolysis conditions and the degree of hydrolysis [21]. It is likely that changes in the physicochemical properties of hydrolysate BW-SPH-F as a result of in vitro gastrointestinal digestion were responsible for the variation in GLP-1 secretion, DPP-IV inhibitory activity and insulin secretion. A study by McLaughlin et al. (2021) reported similar findings as SGID of protein hydrolysates purified from the microalgal *Palmaria palmata* exhibited varying effects on DPP-IV inhibition, insulin secretion in BRIN-BD11 cells and total GLP-1 secretion in GLUTag cells, depending on the protease and hydrolysis conditions employed [22]. 

Secretion of satiety hormones from enteroendocrine cells in response to nutrient signals in the gut is essential for energy homeostasis. Protein hydrolysates which stimulated the in vitro anorexigenic hormone release have also been shown to trigger postprandial physiological responses in vivo, with reductions in food intake, body weight gain and a subjective rate of hunger being reported [7,23]. Protein hydrolysates purified from fish [14,24], dairy [25] and plant [26] origins have been shown to modulate the secretion of various satiety hormones. Cudennec et al. (2012) reported blue whiting (*Micromesistius poutassou*) muscle protein hydrolysates increased the secretion of both active GLP-1 (25-fold) and CCK (30-fold) from STC-1 cells, compared with the basal control (10 mM glucose) at a concentration of 1% *w*/*v* (*p* < 0.05) [7]. Although we also observed increases in active GLP-1 concentrations in STC-1 cells exposed to BWSPHs, CCK and PYY levels were either unaffected or downregulated in vitro. This lack of connection between PYY, CCK and GLP-1 has been reported previously. A study by O’Halloran et al. (2018) also found that a sodium caseinate hydrolysate (10 mg/mL) increased the total GLP-1 secretion from STC-1 cells (*p* < 0.05), but with no effect on CCK or PYY mRNA transcript levels [27]. In order to determine the true physiological effect of BWSPHs-induced satiety hormone secretion, in vivo trials investigating satiety hormone plasma levels and subsequent food intake are necessary. Although there are many advantages of utilising cellular assays for hormone secretion studies, there are also limitations associated with in vitro models compared with in vivo models; in particular, the lack of physiological relevance of in vitro assays [28]. Moreover, a specific limitation of the STC-1 cell line employed herein is its heterogeneity, which can ultimately induce variability in hormone expression; however as recommended by McCarthy et al. (2015), cells were passaged greater than ten times to improve cellular homogeneity [29]. Additionally, large inter-experimental variability was not observed further indicating homogeneity in cells. To further verify the results of this study, the hormone stimulating effect of BWSPHs could be assessed in other murine and human enteroendocrine cells, such as GLUTag cells or NCI-H716 cells, respectively. 

It is possible that the differential modulation of satiety hormones is attributed to specific peptide sequences found within the protein fraction. All BWSPHs tested in this study have previously been characterised by Harnedy-Rothwell et al. (2021) [19]. Physicochemical analysis (degree of hydrolysis, molecular mass distribution and reversed-phase ultra-performance liquid chromatography) demonstrated that all BWSPHs were further hydrolysed during SGID, with the degree of hydrolysis of undigested hydrolysates ranging from 27.82% (BW-SPH-C) to 45.78% (BW-SPH-B), and the degree of hydrolysis of SGID BWSPHs ranging from 55.37% (BW-SPH-C-GI) to 65.23% (BW-SPH-E-GI). BWSPHs were rich in Leu and Arg residues [19], however, the possibility that individual amino acid contents were responsible for the GLP-1 secreting activity of BWSPHs can be discounted as in vitro digestion of BWSPHs, which inhibited proglucagon production and GLP-1 secretion, and increased <1 kDa components and free amino acid levels of all BWSPHs with the exception of Val, thereby suggesting larger peptides were responsible for BWSPHs’ bioactivity, as opposed to the free amino acid content. As discussed previously, the six BWSPHs analysed in this study were prepared with different protease/ protease combinations, therefore, the size and sequence of the peptides generated during hydrolysis differ in each hydrolysate depending on the substrate cleavage site and specificity of the protease employed. In order to identify which peptide(s) are responsible for GLP-1 secretion, it is necessary to identify the peptide sequences in each hydrolysate, thus allowing for peptide sequence homology with known anorectic food-derived peptides listed on peptide databases to be investigated. 

Although GLP-1-secreting L cells increase in number along the length of the gastrointestinal tract, hydrolysis of protein hydrolysates by gastrointestinal proteases can reduce bioactivity. Our data indicates loss of bioactivity post upper gut transit. Hydrolysate BW-SPH-F, which induced the greatest increase in active GLP-1 secretion of the six hydrolysates tested, lost its secretagogue ability when subjected to SGID, indicating its instability towards gastrointestinal proteases. In agreement, Kondrashina et al. (2018) investigated the GLP-1 secretory ability of a casein hydrolysate (10 mg/mL) in STC-1 cells during simulated gut transit and reported a 39% and 51% reduction in GLP-1 secretory capacity following gastric digestion and duodenal digestion, respectively [30]. Loss of bioactivity upon digestion may be a result of an increase in the degree of hydrolysis and the subsequent generation of free amino acids, which are reportedly less effective GLP-1 stimulants [7,31]. Geraedts et al. (2011) also reported that the intestinal digestion of intact codfish protein with intestinal protease trypsin reduced GLP-1 secretion from STC-1 cells (*p* < 0.05) [32]. In contrast to the SGID results in this study, Harnedy and colleagues (2018) demonstrated that in vitro digestion of a different blue whiting protein hydrolysate, prepared with Alcalase 2.4 L and Flavourzyme 500 L, increased GLP-1 secretory activity compared with the non-digested hydrolysate in GLUTag cells (*p* < 0.05), indicating that the release of lower molecular weight peptides from precursor peptides during the digestion process was responsible for the hydrolysates’ secretagogue activity [24]. A significant increase in the degree of hydrolysis of the blue whiting protein hydrolysate following SGID was observed along with an increase in <0.5 kDa components [24]; however, the degree of hydrolysis of the SGID hydrolysate (32.58 ± 0.30%) in the study by Harnedy et al. (2018) was more comparable with the degree of hydrolysis of the undigested BWSPHs in this study [19], again indicating the role of peptides over free amino acid content in blue whiting protein hydrolysate bioactivity. Cudennec et al. (2012) also showed bioactivity maintenance of blue whiting muscle protein hydrolysate in the gut with increased CCK and active GLP-1 plasma levels upon oral administration to male Wistar rats at concentrations of 100 and 250 mg/mL (*p* < 0.05), correlating with a decrease in short term food intake and a decrease in body weight gain [7]. It is possible that the BWSPHs herein may induce GLP-1 production and secretion prior to intestinal digestion through interaction with L-cells located in the duodenum. Harnedy-Rothwell et al. (2021) reported that little to no hydrolysis of BWSPHs occurred during the pepsin (gastric) phase of SGID, and that degradation of BWSPHs predominantly occurred during the simulated intestinal phase upon exposure to intestinal enzymes such as trypsin, chymotrypsin and/or elastase present in Corolase PP [19]. Alternatively, for protection of BWSPHs stability through intestinal delivery, protective coatings such as pH sensitive coatings are available [33]. 

In vitro analysis of the mechanistic actions of BWSPHs further highlighted their ability to influence GLP-1 release. Open-type enteroendocrine cells, which include L cells, where the microvilli of the apical side are in contact with the luminal contents, act as direct sensors of luminal nutrients and non-nutrients to activate hormonal regulators through the activation of G-protein coupled receptors and transporters (ATA2, PEPT1). The activation of G-protein coupled receptors, such as GPR131, stimulates adenylate cyclase, resulting in an increase in the concentration of cAMP within the cells, which is linked to an increase in the intracellular adenosine triphosphate (ATP)/ adenosine diphosphate (ADP) ratio and a subsequent rise in intracellular Ca^2+^ mobilisation [34]. However, in this study, BWSPHs prior to SGID, were observed to increase intracellular Ca^2+^ concentration, without eliciting any effect on intracellular cAMP concentration. It is not uncommon for food-derived protein fractions to stimulate GLP-1 through the activation of calcium pathways [27,35]. The lack of any detectable changes in cellular cAMP levels post BWSPHs incubation in STC-1 cells does not exclude the involvement of a G-protein coupled receptor. Elevation of intracellular Ca^2+^ concentrations can occur through sensing of proteins, peptides and amino acids by enteroendocrine cells which activates a calcium sensing receptor and/or triggers membrane depolarisation via the activation of ion-coupled transporters [34]. 

As the quest for novel proteins with bioactivities continues at pace, it is important that gut barrier health is tracked in line with bioactivity assessment. A differentiated Caco-2/HT-29MTX co-culture was chosen to represent the human intestinal epithelium as absorptive cells (Caco-2), and goblet cells (HT-29MTX) are major cell types of the intestinal epithelial tract [36]. Caco-2 cells secrete pro-inflammatory cytokine IL-6 upon exposure to various inflammatory mediators [37], which can ultimately increase the permeability of Caco-2 cell monolayers, potentially resulting in a homeostatic imbalance of the internal environment [38,39]. No effect of BWSPHs on the viability of intestinal epithelial co-cultured cells was observed, nor were TEER values altered. TEER values remained greater than 700 Ω·cm^2^, indicative of tight junctions [40]. In addition, BWSPHs present in the apical side of co-cultured cells did not induce IL-6 secretion, indicating BWSPHs are unlikely to elicit a sizable immune response in the gut epithelium [41]. Proteins and peptides have been shown to maintain high TEER values and increase tight junction proteins such as occludin and claudin1 [40,42]; however, limited research exists on the protective capacity of proteins derived from fish specifically towards gut health. In agreement with our study, Cinq-Mars et al. (2008) observed that a hake fillet hydrolysate (10 mg/mL) maintained TEER values in Caco-2 cell monolayers above 900 Ω·cm^2^ [43].

To conclude, all BWSPHs upregulated GLP-1 precursor proglucagon mRNA levels and stimulated the secretion of active GLP-1 from STC-1 cells, possibly via intracellular calcium signaling, whilst maintaining the integrity of co-cultured intestinal cells. However, SGID inhibited GLP-1 secretion and proglucagon production, indicating that bioactivity is sensitive to the hydrolytic conditions of the upper gut. BWSPH-derived peptides may require encapsulation or another means of protection to avoid loss of activity during gastrointestinal transition in order to have use as a potential functional food ingredient for weight management. Albeit it would be beneficial to study the effect of simulated gastric digestion, without simulating intestinal digestion, on hydrolysate bioactivity to determine whether bioactive peptides regulate satiety hormone levels, by any degree, in the gut prior to complete proteolysis. Future studies should also identify the peptide(s) responsible for the observed bioactivity of BWSPHs in this study and determine the efficacy of the peptide(s) in vivo. 

## 4. Materials and Methods

### 4.1. Materials

Human Caco-2 cell lines were obtained from the European Collection of Authenticated Cell Cultures (Salisbury, Wilts, UK). Murine STC-1 and human HT-29MTX cell lines were kindly gifted by Dr. Giblin (Teagasc Food Research Centre, Moorepark, Fermoy, Ireland). Mouse IL-6 ELISA kit and Fluo-4 AM calcium indicator were purchased from ThermoFisher Scientific (BioSciences, Dublin, Ireland). Halt Protease and phosphatase inhibitor was purchased from ThermoFisher Scientific (MSC, Dublin, Ireland). Mouse Metabolic Magnetic Bead Panel for active GLP-1 analysis was from Millipore (Cork, Ireland). Foetal bovine serum (FBS) was purchased from Invitrogen (Paisley, Scotland). RNeasy mini kit, DNase digestion kit and the QuantiTect Reverse Transcription kit were from Qiagen (Manchester, UK). LightCycler 480 SYBR Green was from Roche (Roche Products Ireland Limited, Dublin). Forskolin and cAMP enzyme-linked immunosorbent assay (ELISA) kits were from Enzo Life Sciences (Exeter, UK). Cell culture plastics were supplied by Corning incorporated and Cruinn Diagnostics. The remaining chemicals and cell culture reagents were from Sigma Chemical Co. (Dublin, Ireland) unless stated otherwise. BioMarine Ingredients Ireland Ltd. (Lough Egish Food Park, Castleblaney, Co. Monaghan, Ireland) supplied the BWSPHs. 

### 4.2. Sample Preparation 

BioMarine Ingredients Ireland Ltd. (Lough Egish Food Park, Castleblaney, Co. Monaghan, Ireland) supplied the six BWSPHs which were purified from minced blue whiting, as described by Harnedy-Rothwell et al. (2021) [19]. For the generation of hydrolysates BW-SPH-A, BW-SPH-B, BW-SPH-E and BW-SPH-F, fish was solubilised in water at a fish to water ratio of 2:1, whereas a 1.7:1 fish to water ratio was used for the production of hydrolysates BW-SPH-C and BW-SPH-D [19]. The six hydrolysates were prepared with different food-grade, microbial proteases/ protease combinations with enzyme to substrate ratios (E:S) ranging from 0.005% *w*/*w* to 0.900% *w*/*w* and hydrolysis time ranging from 45 to 120 min. The same temperature (50 °C) was used for the generation of the six hydrolysates [19]. Harnedy-Rothwell et al. (2021) completed SGID of the BWSPHs using pepsin (E:S of 2.5% *w*/*w*, 37 °C, pH 2, 90 min) and Corolase PP (E:S of 1% *w*/*w*, 37 °C, pH 7, 150 min) [19]. The study by Harnedy-Rothwell et al. (2021) also details the physicochemical properties of BWSPHs pre- and post-SGID including the degree of hydrolysis, molecular mass distribution and total and free amino acid composition [19]. The degree of hydrolysis ranged from 27.82% to 45.78% in undigested hydrolysates and 55.37% to 65.23% in SGID hydrolysates. The BWSPHs and SGID BWSPHs were prepared directly with Krebs–Ringer buffer, sterile-filtered and stored at −20 °C for STC-1 cell viability and satiety hormone secretion/ production analysis. The BWSPHs and SGID BWSPHs were prepared in Hank’s Balanced Salt Solution (HBSS), sterile-filtered and stored at −20 °C for Caco-2/HT-29MTX co-culture cell viability, barrier integrity and cytokine secretion analysis.

### 4.3. Cell Culture 

STC-1 cells, Caco-2 cells and HT-29MTX cells were grown in 75 cm^2^ tissue culture flasks and cultured in antibiotic free Dulbecco’s Modified Eagles’ Medium (DMEM) supplemented with 10% (*v*/*v*) FBS. STC-1 cells (between passage numbers 23 and 35), Caco-2 cells (between passage numbers 66 and 72) and HT29-MTX cells (between passage numbers 64 and 70) were incubated in an atmosphere of 5% CO_2_ at 37 °C. Media was refreshed every 48 h and cells were passaged every 2–3 days (80–90% confluence). 

### 4.4. Co-Culture

Caco-2 cells (75%) and HT-29MTX cells (25%) in DMEM with 10% FBS was added to polyester permeable-membrane inserts in 12 well plates at a final density of 6.0 × 10^4^ cells/insert. DMEM with 10% FBS (1.5 mL) was added to the basolateral compartment. Cells were differentiated over a 21-day period and apical and basolateral compartments received 500 µL and 1.5 mL of fresh culture media, respectively, every 48 h. 

### 4.5. TEER Measurements

The co-culture monolayer integrity was analysed using a Millicell ERS-2 electrical-resistance system (Millipore, Burlington, MA, USA) on the final day of cell differentiation (day 21). TEER values were measured and co-culture monolayers were washed with HBSS two times. Only monolayers with TEER values exceeding 700 Ω·cm^2^ were used for experiments. BWSPHs were diluted in HBSS to a concentration of 1.0%, *w*/*v* dw and added to the apical compartment (500 µL) for 4 h at 37 °C. Addition of HBSS alone (500 µL) to co-culture monolayers acted as the control. The basolateral compartments received 1.5 mL HBSS. To monitor the integrity of the monolayer, TEER values were recorded before (0 h) and after incubation (4 h). Finally, apical contents were collected and stored at −20 °C prior to cytokine analysis.

### 4.6. Cell Viability 

To test STC-1 cell and Caco-2/HT-29MTX cell viability, STC-1 cells (2 × 10^5^ cells/mL) and a mixture of 75% Caco-2 and 25% HT-29MTX cells (6 × 10^4^ cells/mL) were seeded in 200 µL DMEM, supplemented with 10% FBS, in 96 well plates for 24 h at 37 °C. Well contents were then aspirated and the six BWSPHs (coded BW-SPH-A, -B, -C, -D, -E and -F) and SGID BWSPHs (coded BW-SPH-A-GI, -B-GI, -C-GI, -D-GI, -E-GI and -F-GI) (0–1.0% *w*/*v* dw, 200 µL/well) or DMEM only (control), were added to wells for a further 24 h. The MTT (3-(4,5-dimethylthiazol-2-yl)-2,5-diphenyltetrezolium bromide) assay (MTT I proliferation kit, Roche Diagnostics; Burgess Hill, West Sussex, UK) which consisted of the MTT reagent and solubilisation solution was used to assess cell viability. BWSPHs and DMEM was removed from the wells and replaced with the MTT reagent (10 µL) and DMEM (100 µL) for 4 h at 37 °C. Solubilisation solution (100 µL) was then added to all of the wells and incubation at 37 °C continued overnight. Absorbance (570 nm) was read using a microplate reader (Varioskan TM Flash Multimode Reader, Thermoscientific, Waltham, MA, USA). 

### 4.7. IL-6 Secretion

Caco-2/HT-29MTX co-culture apical samples were subjected to one freeze thaw cycle and the secretion of IL-6 was measured using eBioscience ELISA kits (Ready-SET-Go kit, Thermo Fisher Scientific, Dublin, Ireland). This ELISA kit detects IL-6 at a minimum limit of 4.0 pg/mL

### 4.8. RNA Extraction and Real-Time Reverse Phase Polymerase Chain Reaction (rt-PCR)

Following a 4 h incubation period with Krebs–Ringer buffer (negative control) or BWSPHs (1.0% *w*/*v* dw), STC-1 cells were washed with Phosphate Buffered Saline (PBS) buffer, lysed in 350 µL of lysis buffer and stored overnight at −80 °C. RNA was extracted from the lysate using the RNeasy mini kit and genomic DNA was eliminated from the RNA preparation via on-column DNase digestion, according to manufacturer’s instructions. The Nanodrop 1000 (Thermo Fisher Scientific, Waltham, MA, USA) was used to quantify total RNA with OD 260/280 ratios ranging from 1.93–2.07. RNA (1 μg) was then used to synthesise cDNA. Real-time PCR (LightCycler 96, Roche Diagnostics, Germany) was used to determine proglucagon, CCK and PYY mRNA transcript levels. The various primer sequences, accession codes and annealing temperatures are presented in the table below (Table 1). Amplification efficiencies of primers ranged from 1.87–2.15, using cDNA dilutions of 1:1, 1:10, 1:100 and 1:1000. Each PCR reaction contained 1 μL cDNA, 0.5 μL of forward and reverse primers, 3 μL RNase free water and 5 μL of LightCycler 480 SYBR Green, making a final volume of 10 μL/well. For each sample, the relative amount of target was calculated by the 2^−ΔΔC^_t_ method, where ΔΔC_t_ is [C_t_ (target gene) − C_t_ (36B4)]test condition − [C_t_ (target gene) − C_t_ (36B4)] control condition and C_t_ is the cycle at which the threshold is crossed. 

### 4.9. Active GLP-1 Secretion

STC-1 cells were seeded in 12 well plates at a density of 0.5 × 10^6^ cells/mL in a final volume of 1.25 mL per well. Following a 24 h incubation period, media was removed, and the cells were washed with Krebs–Ringer buffer, which contains 11mM glucose. Cells were acclimatised in Krebs–Ringer buffer (500 µL) for 1 h. Buffer was then replaced by 500 µL of BWSPHs and SGID BWSPHs (1.0% *w*/*v* dw), prepared in Krebs–Ringer buffer, was added to the appropriate wells. Following a 4 h incubation period with Krebs–Ringer buffer (negative control) or BWSPHs, 100X Halt Protease and Phosphatase Inhibitor (5 µL) was added to inactivate endogenous DPP-IV activity. Supernatants were then collected and centrifuged (900 g, 4 °C, 5 min) and stored at −80 °C prior to GLP-1 analysis. The Milliplex Map Kit (Mouse Metabolic Magnetic BeadPanel) and MagPix fluorescent detection system (Luminex, The Netherlands) was used to quantify active GLP-1content in the range of 41–30,000 pg/mL. Intra-assay and inter-assay variation were <10% and <15%, respectively.

### 4.10. Intracellular Ca^2+^ Assay

STC-1 cells were seeded in 96 well plates at a density of 0.25 × 10^6^ cells/mL and final volume of 200 μL per well, for 24 h. After 4 h exposure to 100 μL of BWSPHs (1.0% *w*/*v* dw), SGID BWSPHs (1.0% *w*/*v* dw), carbachol (1mM) (positive control) or Krebs–Ringer buffer (negative control) took place, Fluo-4 AM intracellular calcium probe prepared in Krebs–Ringer buffer (5 μM) was added and cells were further incubated for 1 h at 37 °C. Cell monolayers were then washed 2 times with Krebs–Ringer buffer, which was supplemented with sulfinpyrazone (0.1 mM) to inhibit changes in cytosolic calcium levels. Fluorescence (excitation 488 nm and emission 520 nm) was measured using a microplate reader (Varioskan TM Flash Multimode Reader, Thermoscientific, Waltham, MA, USA).

### 4.11. cAMP Accumulation Assay

STC-1 cells were seeded at 0.5 × 10^6^ cells/mL in a final volume of 1.25 mL per well of a 12 well plate. After 24 h, well contents were removed, and monolayers were washed with Krebs–Ringer buffer, as described above. 3-isobutyl-1-methylxanthine (IBMX) (1 mM) was added to BWSPH sample wells and control wells (positive control was 10 μM forskolin, negative control was Krebs–Ringers buffer) before a 4 h incubation. STC-1 supernatants were then aspirated off and cell lysates were collected after 10 min incubation with 0.2 mL of 0.1 M hydrochloric acid at room temperature. Lysates were then centrifuged (900× *g* for 5 min) and stored at −80 °C before cAMP analysis. A direct cAMP ELISA kit with a minimum detection limit of 0.39 pmol/ mL was used to measure intracellular cAMP levels, and absorbance was read at 405 nm using a microplate reader (Varioskan TM Flash Multimode Reader, Thermoscientific, Waltham, MA, USA).

### 4.12. Statistical Analysis

The data in this study were collected from at least three independent experiments and all results are expressed as the mean ± standard error of the mean (SEM). Significant differences between sample groups and control groups were analysed using a one-way analysis of variance (ANOVA) followed by Dunnett’s test, and Student’s *t*-test was used to determine significant differences between hydrolysates before and after SGID (Prism 5.0, GraphPad Inc. San Diego, CA, USA).

## Figures and Tables

**Table 1:** Primer names, accession numbers, forward and reverse sequences and annealing temperatures.

Primer	Accession Number	Forward Sequence (5′–3′)	Reverse Sequence (5′–3′)	Annealing Temperature
Proglucagon	Z46845.1	CCTTCAAGACACAGAGGAGAAC	GGAGTCGAGGTATTTGCTGTAG	56 °C
CCK	NM_001284508	CTGTCTGCATTTGGCTTGAC	GCCCACTACGATGGGTATTC	55 °C
PYY	NM_145435.1	AACTGCTCTTCACAGACGAC	GTGCCCTCTTCTTAAACCAAAC	55 °C
36B4 (RPLP0)	NM_007475	TGCCACACTCCATCATCAAT	CATCTGATTCCTCCGACTCTT	51 °C

## Data Availability

Data are contained within the article.

## Supplementary Materials

The following are available online at https://www.mdpi.com/article/10.3390/md20020112/s1, Table S1: The effects of blue whiting protein soluble hydrolysates (BWSPHs) pre- and post- simulated gastrointestinal digestion (SGID) on the viability of murine STC-1 cells, Table S2: The effects of blue whiting protein soluble hydrolysates (BWSPHs) pre- and post- simulated gastrointestinal digestion (SGID) on the viability of a Caco-2/HT-29MTX co-culture.
