# Supplementary material for: Blue Whiting (Micromesistius poutassou) Protein Hydrolysates Increase GLP-1 Secretion and Proglucagon Production in STC-1 Cells Whilst Maintaining Caco-2/HT29-MTX Co-Culture Integrity
Source: Mar Drugs. 2022 Jan 31;20(2):112. doi: 10.3390/md20020112 (PMC8877066; doi:10.3390/md20020112)
Supplement: Supplementary file 1 [file marinedrugs-20-00112-s001.zip › Supplementary Table S1.pdf]

Supplementary Table S1. The effects of blue whiting protein soluble hydrolysates (BWSPHs) pre- and post- simulated gastrointestinal digestion (SGID) on the viability of murine STC-1 cells.

	**Cell viability (%)**							
	**0%**	**0.05%**	**0.1%**	**0.25%**	**0.5%**	**0.75%**	0.9%	1.0%
BW-SPH-A	100.0 ± 0.0	110.0 ± 6.1	105.9 ± 3.8	99.0 ± 0.5	99.0 ± 4.3	99.9 ± 5.8	97.8 ± 5.0	88.1 ± 7.0
BW-SPH-A-GI	100.0 ± 0.0	105.8 ± 10.3	114.0 ± 11.5	116.4 ± 12.3	118.5 ± 14.7	111.3± 10.5	116.4 ± 13.2	96.4 ± 4.8
BW-SPH-B	100.0 ± 0.0	105.5 ± 5.2	101.8 ± 4.1	102.0 ± 2.4	96.8 ± 3.6	103.3 ± 5.7	101.6 ± 5.6	93.3 ± 2.3
BW-SPH-B-GI	100.0 ± 0.0	127.3 ± 4.1	118.6 ± 4.4	119.3 ± 2.4	129.1 ± 5.4	127.0 ± 7.6	123.3 ± 8.8	96.7 ± 5.1
BW-SPH-C	100.0 ± 0.0	107.6 ± 8.0	106.6 ± 5.3	106.3 ± 7.1	99.9 ± 7.3	108.6 ± 8.3	103.8 ± 6.4	92.2 ± 8.5
BW-SPH-B-GI	100.0 ± 0.0	123.3 ± 0.5	117.1 ± 3.5	128.3 ± 2.9	128.2 ± 5.3	129.5 ± 7.8	122.7 ± 9.8	93.0 ± 3.1
BW-SPH-D	100.0 ± 0.0	93.8 ± 3.2	84.8 ± 4.7	86.8 ± 6.1	84.5 ± 7.9	83.0 ± 7.9	82.4 ± 7.2	82.4 ± 9.0
BW-SPH-D-GI	100.0 ± 0.0	122.4 ± 7.5	114.3 ± 5.9	121.1 ± 4.4	124.6 ± 7.5	116.0 ± 7.0	122.6 ± 7.8	94.3 ± 0.8
BW-SPH-E	100.0 ± 0.0	106 ± 5.4	104.4 ± 4.2	100.8 ± 4.1	97.8 ± 5.0	99.2 ± 2.9	99.5 ± 4.6	86.8 ± 0.7
BW-SPH-E-GI	100.0 ± 0.0	122.5 ± 5.2	117.7 ± 5.5	124.8 ± 5.6	124.2 ± 9.4	122.0 ± 8.2	113.8 ± 5.3	94.4 ± 3.7
BW-SPH-F	100.0 ± 0.0	103.7 ± 2.0	100.2 ± 2.4	105.8 ± 2.2	95.7 ± 2.9	98.7 ± 6.3	95.6 ± 1.4	84.4 ± 0.3
BW-SPH-F-GI	100.0 ± 0.0	105.7 ± 8.7	106.4 ± 10.5	104.1 ± 8.4	106.8 ± 10.9	103.1 ± 6.4	116.0 ± 6.6	103.8 ± 5.0

Murine STC-1 cells (2 x 10^5 cells/mL, 200 μL/well) were supplemented with increasing concentrations (0-1.0%, w/v dw) of BWSPHs (BW-SPH-A to BW-SPH-F) and SGID BWSPHs (BW-SPH-A-GI to BW-SPH-F-GI) for 24 hours. Cell viability was determined by the MTT assay and expressed as a percentage of the untreated control. Data are the mean ± SEM of three independent experiments. Significance was measured using ANOVA followed by Dunnett's test.
