# Supplementary material for: Blue Whiting (Micromesistius poutassou) Protein Hydrolysates Increase GLP-1 Secretion and Proglucagon Production in STC-1 Cells Whilst Maintaining Caco-2/HT29-MTX Co-Culture Integrity
Source: Mar Drugs. 2022 Jan 31;20(2):112. doi: 10.3390/md20020112 (PMC8877066; doi:10.3390/md20020112)
Supplement: Supplementary file 1 [file marinedrugs-20-00112-s001.zip › Supplementary Table S2.pdf]

Supplementary Table S2. The effects of blue whiting protein soluble hydrolysates (BWSPHs) pre- and post- simulated gastrointestinal digestion (SGID) on the viability of a Caco-2/HT-29MTX co-culture.

	**Cell viability (%)**							
	**0%**	**0.05%**	**0.1%**	**0.25%**	**0.5%**	**0.75%**	0.9%	1.0%
BW-SPH-A	100.0 ± 0.0	95.8 ± 8.5	104.4 ± 6.5	111.5 ± 11.1	114.5 ± 12.0	113.2 ± 4.0	111.6 ± 9.0	110.1 ± 6.6
BW-SPH-A-GI	100.0 ± 0.0	102.5 ± 6.1	107.1 ± 9.3	109.0 ± 7.2	107.8 ± 7.6	104.6 ± 1.8	106.1 ± 3.3	102.7 ± 5.7
BW-SPH-B	100.0 ± 0.0	100.3 ± 3.0	102.2 ± 4.6	99.2 ± 4.6	102.9 ± 6.4	100.7 ± 1.5	101.0 ± 3.1	109.0 ± 14.9
BW-SPH-B-GI	100.0 ± 0.0	111.0 ± 4.5	109.7 ± 5.5	104.5 ± 2.6	110.6 ± 3.7	111.0 ± 3.6	114.0 ± 6.2	107.5 ± 7.9
BW-SPH-C	100.0 ± 0.0	104.7 ± 2.8	107.2 ± 7.1	102.5 ± 2.7	103.9 ± 10.6	97.5 ± 8.8	107.3 ± 5.4	102.3 ± 8.3
BW-SPH-B-GI	100.0 ± 0.0	93.4 ± 6.5	103.3 ± 5.9	107.2 ± 4.6	102.0 ± 6.7	105.4 ± 4.4	106.3 ± 3.1	96.6 ± 5.9
BW-SPH-D	100.0 ± 0.0	102.7 ± 8.1	99.1 ± 10.5	106.4 ± 8.6	102.4 ± 12.2	99.9 ± 10.0	102.2 ± 10.3	110.7 ± 2.9
BW-SPH-D-GI	100.0 ± 0.0	93.3 ± 9.1	105.8 ± 1.5	104.2 ± 6.1	106.8 ± 3.1	108.2 ± 5.1	113.9 ± 5.9	98.0 ± 5.5
BW-SPH-E	100.0 ± 0.0	100.2 ± 7.7	103.9 ± 8.9	105.5 ± 6.5	108.6 ± 9.0	100.2 ± 5.1	102.4 ± 4.7	107.7 ± 7.3
BW-SPH-E-GI	100.0 ± 0.0	109.8 ± 4.4	114.9 ± 3.0	109.3 ± 4.7	118.3 ± 7.5	116.9 ± 6.0	122.5 ± 3.8	108.4 ± 9.8
BW-SPH-F	100.0 ± 0.0	96.4 ± 8.9	97.0 ± 6.2	94.4 ± 4.9	99.7 ± 12.0	102.8 ± 7.9	97.8 ± 9.3	112.0 ± 8.0
BW-SPH-F-GI	100.0 ± 0.0	112.3 ± 11.4	124.0 ± 8.0	116.2 ± 7.6	123.5 ± 10.4	117.9 ± 9.9	124.8 ± 4.0	114.2 ± 2.5

Caco-2/HT-29MTX co-culture (6 x 10^4 cells/mL, 200 µL/well) were supplemented with increasing concentrations (0-1.0%, w/v dw) of BWSPHs (BW-SPH-A to BW-SPH-F) and SGID BWSPHs (BW-SPH-A-GI to BW-SPH-F-GI)in DMEM media for 24 hours. Cell viability was determined by the MTT assay and expressed as a percentage of the untreated control ‘0%’ (DMEM only). Data are the mean ± SEM of three independent experiments. Significance was measured using ANOVA followed by Dunnett’s test.
